# Neuromuscular abnormality and autonomic dysfunction in patients with cerebrotendinous xanthomatosis

**DOI:** 10.1186/1471-2377-11-63

**Published:** 2011-05-31

**Authors:** Shu-Fang Chen, Nai-Wen Tsai, Chung-Chih Chang, Cheng-Hsien Lu, Chi-Ren Huang, Yao-Chung Chuang, Wen-Neng Chang

**Affiliations:** 1 Department of Neurology, Kaohsiung Chang Gung Memorial Hospital and Chang Gung University College of Medicine, Kaohsiung, Taiwan; 2 Department of Biological Science, National Sun Yat-Sen University, Kaohsiung, Taiwan

## Abstract

**Background:**

Cerebrotendinous xanthomatosis (CTX) is a rare lipid-storage disease. Neuromuscular abnormality and autonomic system (ANS) dysfuction in CTX are rarely examined in large-scale studies in the literature. We studied the peripheral nervous system, myopathology, and autonomic system of four CTX patients and performed a literature review of the reported CTX patients with peripheral neuropathy.

**Methods:**

Four biochemically and genetically confirmed CTX patients, belonging to two families, were included for study and all received nerve conduction study (NCS), muscle biopsy for histopathologic and ultrastructural study, skin biopsy for intraepidermal nerve fiber (INEF) density measurement, autonomic testings including sympathetic skin response, R-R interval variation and head-up tilt test using an automated tilt table to record the changes of blood pressure and heart rate in different postures. The Q-Sweat test was also applied for the detection of sweat amount and onset time of response. The clinical characteristics, study methods and results of 13 studies of peripheral neuropathy in CTX patients in the literature were also recorded for analysis.

**Results:**

The results of NCS study showed axonal sensory-motor polyneuropathy in three CTX cases and mixed axonal and demyelinating sensor-motor polyneuropathy in one. The myopathological and histopathologic studies revealed mild denervation characteristics, but the ultrastructural study revealed changes of mitochondria and the membranous system, and increased amounts of glycogen, lipofuscin and lipid deposition. The ANS study revealed different degrees of abnormalities in the applied tests and the INEF density measurement showed small fiber neuropathy in three of the four CTX patients. The literature review of peripheral neuropathy in CTX revealed different types of peripheral neuropathy, of which axonal peripheral neuropathy was the most common.

**Conclusions:**

Peripheral neuropathy, especially the subtype of axonal sensori-motor neuropathy, is common in patients with CTX. Evidence of lipid metabolic derangement in CTX can be reflected in ultrastructural studies of muscles. With an adequate multi-parametric evaluation, a high incidence of ANS abnormalities can be seen in this rare lipid-storage disease, and a high incidence of small fiber involvement is also reflected in the IENF density measurement of skin biopsies.

## Background

Cerebrotendinous xanthomatosis (CTX), a rare autosomal recessive cholestanol lipidosis, is caused by a deficiency of the gene encoding the mitochondrial sterol 27-hydroxylase (CYP27A1) [1-4] and subsequently results in an abnormal deposition of cholestanol and cholesterol in various organs. The major clinical presentations of CTX are juvenile cataracts, tendinous xanthomatosis and various neurological symptoms including dementia, pyramidal signs and cerebellar ataxia [1-4]. There have been case reports and small-scale studies on peripheral neuropathy in CTX using different measuring methods [5-17]. However, the state of the autonomic nervous system (ANS) [14,16] and myopathologic features [15,18] in CTX have rarely been reported, and different results have been noted among these limited studies. In this study, we reported the abnormalities of neuromuscular and ANS systems of four CTX patients and performed a literature review of the reported CTX-related peripheral neuropathy.

## Methods

### Patients

The clinical data of the four included CTX cases are listed in Table 1. All four cases (Cases I-1, I-2, II-1 and II-2) had an elevation of serum cholestanol at presentation, with initial levels ranging from 24.7 to 46.8 ug/ml (mean = 34.3 ug/ml, normal value = 3.37 ± 1.55 ug/ml). The diagnosis of CTX was made in 1991 in Family I, and 2004 in Family II. The clinical and neuroimaging features, and results of biochemical and genetic studies of these four CTX cases have been previously reported [19-21]. All four cases received chenodeoxycholic acid (CDCA) treatment (750 mg/day) from the time of diagnosis. There were three CTX siblings in Family I, however one died before this study, and therefore only two were included in this study. This retrospective study was approved by the ethics committee of Chang Gung Memorial Hospital (IRB 99-3615B). Cases I-1 and I-2 (Family 1) and Cases II-1 and II-2 (Family II) have consented to publication of their clinical, laboratory and genetic data on March 30, 2006 and October 12, 2004, respectively.

**Table 1:** Clinical data of the four cerebrotendinous xanthomatosis (CTX) patients

Family	I		II	
Case No	1	2	1	2
Gender	F	M	M	M
Age (years)	54	50	31	29
Age at CTX diagnosis (years)	37	32	24	23
Disease duration at neuromuscular study (years)	49	45	21	17
Body height (cm)	143	155	164	175
Body weight (kg)	45	50	74	114
Genetic mutation (*CYP 27 *gene)	Homozygous: 305delC (exon 2)		Heterozygous: 1333C > T (exon 8) and IVS 7+1G > A (intron 7)	
Clinical manifestation				
Tendinous xanthomatosis	+	+	+	+
Cataracts	+	+	+	+
Osteoporosis with multiple fractures	+	+	-	-
Mental retardation	+	+	+	+
Cerebellar sign	+	+	+	+
Pyramidal sign	+	+	+	+
Parkinsonism	+	+	-	-
Glove-stock paresthesia	-	-	-	-
Hyporefelxia	-	-	-	-
Distal foot muscle atrophy	-	-	-	-
Posture related problems	-	-	-	-
Sphincter problems	-	-	-	-
Sweating problems	-	-	-	-

No = number; + = present; - = absent; F = female; M = male

### Nerve conduction study and electromyography study

Nerve conduction studies (NCS) studies were performed using a VikingSelect system (Nicolet Biomedical Inc. Madison, USA). All testing was done in the same room and with the same temperature conditions, and the skin temperature was kept ≧32°C. The studied nerves included sural, tibial, peroneal, superficial radial, medial plantar, median and ulnar nerves. Amplitudes of the sensory action potential and compound muscle action potential as well as nerve conduction velocity, F wave response, distal latency and soleus-H reflex were recorded. In this study, the referential data for Chinese were cited from the report of Lin et al. [22]. In electromyographic (EMG) studies, the proximal and distal muscles of the upper and lower limbs were sampled.

### Muscle biopsies

Muscle biopsies from the vastus lateralis of quadriceps were obtained with a BergstroÈm needle in all four CTX cases. Both histopathological and ultrastructural studies were processed according to standard proctocols [23-25]. The specimens processed for light microscopic investigations, were stained with haematoxylin/phloxine, ATPase, succinate dehydrogenase, cytochrome oxidase, Sudan black, periodic acid-Schiff and acid phosphatase. All muscle specimens were also prepared for ultrastructural examinations.

### Skin biopsies and quantitation of skin innervation

A punch skin biopsy of 3-mm in diameter was taken from the right distal leg 10 cm proximal to the lateral malleolus under local anesthesia with 2% lidocaine following the established protocol [26]. The sampled skin tissue was fixed in paraformaldehyde overnight. Sections 50 μm perpendicular to the dermis were immunostained with antiserum to protein gene product 9.5 (PGP 9.5, 1: 1000; UltraClone, Isle of Wight, UK) and the reaction product was demonstrated using chromogen SG (Vector Laboratories).

Epidermal innervation was quantified according to established criteria in a coded fashion [27]. The observers were blinded to the clinical information. PGP 9.5 (+) nerves in the epidermis of each skin section were counted at a magnification of 40× with a BX40 microscope (Olympus, Tokyo, Japan). The length of the epidermis along the upper margin of the stratum corneum in each skin section was measured with ImageJ version 1.43 software (Image Processing and Analysis in Java, National Institutes of Health, Bethesda, MD: http://rsbweb.nih.gov/ij/download.html). The intraepidermal nerve fiber (IENF) density was expressed as the number of fibers/mm of epidermal length. In the distal leg, normative values (mean ± SD, 5th percentile) of IENF are 11.16 ± 3.70 (5.88) fibers/mm for subjects aged < 60 years and 7.64 ± 3.08 (2.50) fibers/mm for subjects aged ≧ 60 years, and age- and gender-matched controls were retrieved from the previously described database [28].

### Autonomic function assessments

Sympathetic skin response (SSR) and R-R interval variation (RRIV) were performed using a VikingSelect system (Nicolet Biomedical Inc. Madison, USA). RRIV is defined as heart rate (HR) variability in deep breathing test (patients are asked to breath at a rate of 6 breaths/min ) as maximus HR -minimus HR/mean HR, and it is used to measure the cardiac parasympathetic activity [29]. Head-up tilt (HUT) tests for orthostatic blood pressure (BP) and heart rate (HR) changes were performed on an automated tilt table. Patients were first placed in a supine position and then tilted to 70 degrees. Data recorded using a continuous monitor (Finapres BP monitor 2300, Finameter Pro, Ohmeda; Englewood, OH, USA) included systolic, diastolic, and mean BP continuously in serial recordings at 0, 1, 2, 3, and 5 minutes after tilting up, and at the third minute after lying back. In this study, "orthostatic hypotension" was defined as a reduction in a systolic BP of ≧ 20 mmHg or a diastolic BP of ≧ 10 mmHg within three minutes of upright tilt to an angle of ≧ 60° or standing. We used a "30:15 ratio" which may denote the cardiac parasympathetic activity [30], to assess this physiological response by measuring the ratio of the RR interval at beat 30 while standing to the RR interval at beat 15. The slope of the regression line between the R-R interval and systolic BP values was computed for each sequence, and taken as a measurement of baroreflex sensitivity (BRS, ms/mmHg) which may measure both sympathetic and parasympathetic activities [31] in this HUT study [32].

### Q-Sweat recordings

The Q-Sweat recording was performed at the same room temperature (21-23°C), room humidity (56-58%) and skin temperatures over the tested sites (> 34°C). All four CTX cases underwent left limb Q-Sweat recordings using the standard regional sites (forearm, proximal leg, distal leg, and foot) with a Q-Sweat device (WR Medical Electronics Co, Stillwater, Minnesota, USA) by the evoke of acetylcholine solution (10% w/v). Sweat amount and onset time of response were displayed using TestWorks software (WR Medical Electronics Co., Stillwater, Minnesota) [33]. In addition, 90 sex- and age-matched healthy subjects from the normative database acted as control for this test. The human ethics committee of Chang Gung Memorial Hospital approved the study (IRB98-0805B).

### Reported CTX patients with peripheral neuropathy in the literature

For a better delineation of peripheral neuropathy features in CTX patients, the clinical data and described types of peripheral neuropathy reported in the literature [5-17] were included for analysis.

## Results

### NCS and EMG

The results of the NCS study of the four CTX cases showed a mild axonal sensory-motor polyneuropathy characterized by a decreased amplitude in sensory and motor nerves, and an absent response in the sural nerves in Cases I-1, I-2 and II-2, and mixed axonal and demyelinating sensory-motor polyneuropathy characterized by slowed nerve conduction velocity, prolonged F response and absent response in sural nerves in Case II-1 (Table 2). In EMG studies, only denervation changes characterized by decreased recruitment patterns and increased polyphasic waves in the distal muscles were detected.

**Table 2:** Nerve conduction study findings of the four cerebrotendinous xanthomatosis patients

Case		Median		Ulnar		Superficial radial	Peroneal	Tibial	Sural	H-reflex (N < 27.6)
		Motor	Sensory	Motor	Sensory					
I-1	R DL (ms)	4.4 (N < 3.6)	2.8	2.9 (N < 2.8)	2.2	1.9	4.0 (N < 4.2)	2.7 (N < 4.9)	Absent	31.0
	L DL (ms)	4.4	3.0	2.5	2.9	1.7	4.0	3.7	Absent	30.4
	R CV (m/s)	50 (N > 57.8)	50 (N > 51.7)	50 (N > 61.4)	56 (N > 56.3)	63 (N > 50)	39 (N > 48.6)	45 (N > 50.5)	Absent (N > 47.4)	
	L CV (m/s)	49	47	54	49	71	42	40	Absent	
	R Amp	7.6 (N > 11.9)	47 (N > 33)	11 (N > 11.7)	32 (N > 26.1)	23 (N > 15)	4.1 (N > 6.0)	5.5 (N > 13.1)	Absent (N > 12.5)	
	L Amp	7.1	21	8.1	38	33	4.5	9.6	Absent	
	R F waves	26.8-0.7 (N < 25.4)		27.0-0.8 (N < 25.0)			49.8-0.4 (N < 44.7)	50.2-0.8 (N < 45.0)		
	L F waves	27.9-0.8		26.4-1.0			47.4-0.4	50.4-0.8		
I-2	R DL (ms)	4.4	2.7	3.2	2.6	2.1	4.2	5.0	Absent	32.4
	L DL (ms)	4.0	2.8	3.0	2.2	1.8	3.9	3.3	Absent	32.6
	R CV (m/s)	49	53	54	46	57	39	41	Absent	
	L CV (m/s)	51	50	51	54	67	41	40	Absent	
	R Amp	8.6	23	7.3	26	34	2.9	10.5	Absent	
	L Amp	9.2	31	8.8	13	60	3.8	6.7	Absent	
	R F waves	30.0-0.5		28.8-1.0			50.5-1.0	51.8-1.0		
	L F waves	28.3-1.0		28.4-1.0			50.1-0.6	53.9-1.0		
II-1	R DL (ms)	4.3	2.8	3.0	2.4	2.1	Absent	4.5	Absent	38.4
	L DL (ms)	4.1	3.0	2.8	2.5	2.0	4.9	5.1	Absent	37.5
	R CV (m/s)	44	50	42	50	56	Absent	32	Absent	
	L CV (m/s)	46	47	43	48	60	30	34	Absent	
	R Amp	10.3	42	11.5	20	25	Absent	4.0	Absent	
	L Amp	11.7	47	9.9	29	32	2.4	2.6	Absent	
	R F waves	32.6-1.0		32.1-1.0			Absent	70.6-0.6		
	L F waves	30.4-0.9		30.4-1.0			79.2-0.2	75.7-0.9		
II-2	R DL (ms)	4.0	2.5	2.8	2.0	1.7	4.4	3.4	Absent	31.9
	L DL (ms)	4.2	2.7	3.0	2.3	1.7	3.8	3.8	Absent	31.6
	R CV (m/s)	52	56	58	60	71	39	41	Absent	
	L CV (m/s)	52	52	50	53	71	43	39	Absent	
	R Amp	8.5	29	6.6	38	16	3.2	4.7	Absent	
	L Amp	7.4	50	6.7	40	29	4.7	2.0	Absent	
	R F waves	29.8-1.0		30.4-0.8			54.2-0.3	53.3-1.0		
	L F waves	29.6-1.0		31.6-0.9			50.9-0.8	56.5-0.9		

N = normal; DL = distal latency; CV = conduction velocity; Amp = amplitude (mV in motor nerves and μV in sensory nerves); F waves = minimal latency-persistent rate; H-reflex = soleus H-reflex in minimal latency; AT = anterior tibialis; FDI = 1st dorsal interosseous muscle; R = right; L = left

### Muscle biopsies

**Figure 1:**
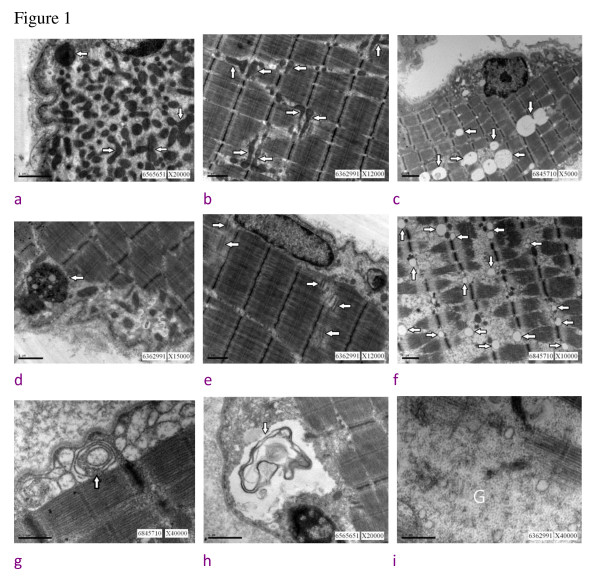
**Summarized muscle ultrastructural findings of the four cerebrotendinous xanthomatosis cases**. 1a. subsacrolemmal accumulation of mitochondria (arrow), (bar = 1 μm); 1b. mitochondria change in shape and enlarged in size (arrow), (bar = 1 μm); 1c. increased amount of lipid droplets (arrow), (bar = 2 μm); 1d. lipofuscin (arrow), (bar = 1 μm); 1e. triads proliferation (arrow), (bar = 1 μm); 1f. swollen sacroplasmic reticulum (arrow), (bar = 1 μm); 1g. laminated bodies (arrow), (bar = 0.5 μm); 1 h. membranous-like debris (arrow) in the subsacrolemmal space, (bar = 1 μm); 1i. increased amount of glycogen (marked as G, bar = 0.5 μm).

In light microscopic examination, no histopathologic features suggesting myopathic change were noted, but small grouping denoting slight neurogenic changes was observed. The findings of the ultrastructural study are listed in Table 3 and demonstrated in Figure 1.

**Table 3:** Ultrastructural features of myopathology of the four cerebrotendinous xanthomatosis patients

	I-1	I-2	II-1	II-2
**Changes of mitochondria**				
subsacrolemmal accumulation	+++	+++	++	+
change in size and shape	+++	++	++	+
increase in amount	+++	++	++	+
**Changes of membranous system**				
triads proliferation, swollen	-	++	+	-
increased amount of sacroplasmic reticulum	+	++++	-	+++
laminated bodies	-	-	-	++
membranous debris	+++	++	++	++
**Increased amount of lipid droplets**	+	++	+++	++++
**Increased amount of lipofuscin**	+	+	+	++
**Increased amount of glycogen**	++	++	+	++

+ = positive; - = negative; the number of "+" = different amounts

### Quantitation of skin innervations

The IENF densities were as follows: 3.19 fibers/mm in Case I-1, 6.14 fibers/mm in Case I-2, 5.45 fibers/mm in Case II-1 and 5.13 fibers/mm in Case II-2. This result showed a decrease of IENF in Cases I-1, II-1 and II-2.

### Results of autonomic testings (Table 4)

In RRIV measurement, decreased value was found in Case I-1. In SSR measurement, no abnormal responses were detected on either the soles or palms of these four cases. The results of the HUT test showed: 1) no abnormal orthostatic HR or BP changes in all four cases; 2) abnormally decreased values in Cases I-1 and I-2 in the measurement of the "30:15 ratio"; and 3) negative and decreased values in Cases I-1 and I-2 in BRS measurement.

In the Q-Sweat test, compared with the data of normal control (Table 4), sweat output showed a reduction of sweat volume only in the forearm and foot of Case II-1. With a relative comparison, the sweat volumes were as follows: foot sweat volume < 1/3 of the forearm value in Case I-1; foot and proximal leg < 1/3 of the forearm value in Case I-2; and forearm and foot volume < 1/3 of the proximal leg value in Case II-1.

**Table 4:** Autonomic findings

		I-1	I-2	II-1	II-2
RRIV (%) (N > 15)		3.53	15.7	24.2	25.4
Head-up tilt test		NR	NR	NR	NR
BR sensitivity (msec/mmHg)		-1.9	-1.4	2.3	0.7
30:15 ratio (N > 1.03)		1.03	0.95	1.11	1.37
SSR					
onset latency (ms)	R palm	1320	1700	1440	1720
	L palm	1400	1700	1400	1770
	R sole	2300	2280	2060	2280
	L sole	2290	2280	2250	2280
amplitude (μV)	R palm	2652	1569	978	1314
	L palm	2198	921	869	1099
	R sole	315	159	61	688
	L sole	399	239	74	562
Q-Sweat test					
onset latency (seconds)	Forearm	137	219	185	275
	Proximal leg	217	194	165	128
	Distal leg	165	148	168	136
	Foot	165	203	189	122
total volume (μl)	Forearm	0.150	0.298	0.015	0.450
	Proximal leg	0.134	0.063	0.199	1.283
	Distal leg	0.183	0.108	0.294	1.409
	Foot	0.029	0.095	0.030	0.700
skin temperature (°C)	Forearm	36.5	36.4	36.5	35.4
	Proximal leg	35	35.8	36.4	35.7
	Distal leg	35.6	36.3	34.9	35.3
	Foot	35.7	35.2	34.7	35.4
room temperature (°C)		23	21	21	21
room humidity (%)		56	57	58	58

RRIV: R-R interval variation; NR: normal blood pressure (BP) and heart rate (HR) response in head-up tilt test: no abnormal change in BP and HR; N: normal; SSR: sympathetic skin response; Q-Sweat: quantitative sweat measurement.

In Q-Sweat test, the total sweat volume (median (min, max) for female normal control were 0.495 (0.02, 3.814) (forearm), 0.505 (0.06, 4.49) (proximal leg), 0.389 (0.015, 4.914) (distal leg) and 0.203 (0.014, 0.802) (foot), and for male normal control were 1.079 (0.02, 3.814) (forearm), 1.091 (0.029, 2.734) (proximal leg), 1.107 (0.043, 2.902) (distal leg) and 0.585 (0.041, 1.718) (foot).

### Reported CTX patients with peripheral neuropathy: summary of clinical features (Table 5)

In the literature review, we found 13 reports [5-17] that had studied peripheral neuropathy of CTX patients. The clinical and laboratory data of these studies were included for analysis.

## Discussion

### Peripheral Nerve Abnormality

CTX is an uncommon lipid storage disease, and its associated neuromuscular abnormalities have rarely been examined in large-scale studies. The reported CTX cases with peripheral neuropathy [5-17] used different measurement methods (NCS and/or EMG and/or sural nerve biopsy and/or muscle biopsy) for assessment and confirmation, and also listed differently-described types of peripheral neuropathy (Table 5). Because of these differences in clinical evaluation and study results, a matter of debate in the pathologic findings and etiologies of peripheral neuropathy in CTX were also elicited among these reports [5-17]. Mondelli *et al. *[13], used electrophysiologic studies including NCS, and they found that eight CTX patients had a moderate sensori-motor neuropathy of demyelinating type, but this finding was not further confirmed by pathological study of nerve. Argov *et al. *[9] also noted a demyelinating process in a study of peripheral neuropathy in three CTX patients. In another study by Verrips *et al. *[15] using NCS and EMG studies, seven of their included CTX cases had a peripheral neuropathy (five with axonal type, one with mixed axonal and demyelinating type, and one unspecified), two a normal study and one with myopathic findings. In our study, different degrees of axonal sensory-motor polyneuropathy in three cases and mixed axonal and demyelinating sensori-motor polyneuropathy in one case were found, and their myo-histopathologic features also reflected this neuropathic process showing denervation features. Our study results are consistent with those of Verrips *et al. *[15] in that axonal degeneration was concluded to be the predominant process of neuropathy in CTX, although in some of their patients, features of chronic demyelination and remyelination were also found [15]. In pathologic studies of peripheral nerves, Voiculescu *et al. *[10], Donaghy *et al. *[11] and Wang *et al. *[17] found the deposition of lipid in Schwann cells in CTX patients with neuropathy, while this pathologic change was not noted in the studies of Katz *et al. *[8], Argov *et al. *[9] and Verrips A *et al.*, [15]. Besides our four cases, another 12 CTX patients [15-17] with a peripheral neuropathy had a genetic analysis, but different mutation patterns were noted. This lack of correlation between the genotype and pathogenesis of peripheral neuropathy or other phenotypes was noted in the studies of Verrips *et al. *[15,34].

**Table 5:** Clinical and laboratory data of the reported cerebrotendinous xanthomatosis patients with peripheral neuropathy [5-17]

Reference No/ year	Total case No/gene mutation/ age or range of age (y/r)	Described type of neuropathy	Nerve conduction study	Sural nerve biopsy	Muscle biopsy
[5]/ 1079	4/ND/12-27	S-M pnp	D	ND	ND
[6]/1979	1/ND/25	ND	D	D	ND
[7]/1984	4/ND/35-43	S-M pnp	D	D	D
[8]/1985	1/ND/50	S-M pnp	D	D	ND
[9]/1986	3/ND/14-30	demyelinated S-M pnp	D	D	ND
[10]/1987	1/ND/29	S-M pnp	D	D	D
[11]/1990	1/ND/30	S-M pnp	D	D	ND
[12]/1991	1/ND/22	Axonal pnp	D	D	ND
[13]/1992	10/ND/26-44	Mixed S-M pnp (2) and demyelinating S-M pnp (8)	D	ND	ND
[14]/1995	1/ND/34	Mixed	D	ND	ND
[15]/2000	10/D^a^/24-54	Axonal pnp (5), mixed pnp (1), pnp (1)	D	D	D
[16]/2007	1/D^b^/47	Polyneuropathy	D	ND	ND
[17]/2007	1/D^c^/42	S-M polyneuropathy	D	D	ND

No = number; ND = not determined; D = determined; S-M = sensori-motor; pnp = peripheral neuropathy;

^a ^= mutations on both alleles of the *CYP 27 *gene in all 10 patients (Table 1 of [15])

^b ^= mutations 1016C > T (T339M) and 1183C > T (R395C) in the *CYP21A2 *gene

^c ^= mutations 379C > T (R94W) and 1420 C > T (R441W) in the *CYP27A1 *gene

### Muscle Abnormality

In the myopathologic study, mild neurogenic change was the main finding of our four CTX cases, and this denervation feature was also found in other related reports of CTX patients with peripheral neuropathy [7,10,15,18]. In the ultrastructual study of muscles, the main abnormal findings included changes of the mitochondria and membranous system, and an increased amount of lipid droplets, lipofuscin and glycogen (Table 3). These ultrastructual findings are consistent with those reported by Verrips *et al. *[15] and Federico *et al. *[18]. Based on these ultrastructural changes, Federico *et al. *[18] concluded that: the primary alteration of lipid metabolism in CTX might lead to an impairment of the energy processes of muscle and subsequent mitochondrial morphological change. However, all these ultrastructural changes are nonspecific for CTX and can be found in other muscle disorders or can even be a normal constituent of human skeletal muscles [15,24,25]. Compared with the nervous system, muscles are not a preferable site for cholestanol deposition [35], but all these ultrastructural changes can be attributed to an abnormal metabolic state in CTX which is reflected by the abnormality of the membrane system and abnormal deposition of lipid droplets, lipofuscion and glycogen as shown in this study.

### ANS Abnormality

In the literature, the state of ANS in CTX has only been described in two case reports [14,16]. In the report of Geraldes *et al. *[16], no involvement of the ANS in a CTX case with severe neuropathy was found. However in the report of Arpa *et al. *[14], an opposite result was found, showing a postganglionic cholinergic failure in a CTX patient with somatic peripheral neuropathy. In this study, we arranged multiparametric evaluation (SSR, RRIV, HUT study for orthostatic BP and HR recording, and Q-Sweat recording) to assess the functional state of ANS of the four CTX patients. The evaluation results revealed different degrees of laboratory evidence of ANS abnormalities in three of the four CTX patients, in which both sympathetic and parasympathetic systems were involved (Table 4). Despite these different degrees of laboratory abnormalities were demonstrated, they were not reflected fully in clinical blood pressure and heart rate changes in these four cases. In the meanwhile, there was also a mismatch in the inter-lab data such as the finding of SSR and Q-Sweat test. These mismatches need further verification by large-scale study.

This ANS abnormality can be partially reflected pathologically by the findings of skin biopsy IENF measurement, which is a reliable tool with high diagnostic specificity and good sensitivity for small fiber disease [36-38]. In a study conducted by Schuller *et al. *[39], a comparison of the three small fiber systems revealed that functionally different systems were damaged independently, and isolated affection of each fiber type was frequently observed. Therefore, biopsy results must be interpreted in conjunction with neurologic findings and laboratory results, including complementary testing of several small somatic systems and ANS. But again, as shown in this study, there is a discrepancy between the finding of IENF density measurement and Q-Sweat test. This mismatch of the inter-lab data also needs further large scale study for verification. In this study, IENF density measurement revealed a relatively high incidence (75%, 3/4) of small fiber involvement in CTX, and this figure of incidence is as high as those neuropathies with a small-fiber sensory neuropathy [40-43].

## Conclusion

Peripheral neuropathy, especially the subtype axonal sensori-motor neuropathy, is common in patients with CTX. Evidence of lipid metabolic derangement in CTX can be reflected in ultrastructural studies of muscles. With an adequate multi-parametric evaluation, a high incidence of ANS abnormality can be seen in this uncommon lipid-storage disease, and a high incidence of small fiber involvement is also reflected in the IENF density measurement of skin biopsies. This study is limited by its small case number, but it may shed light on further studies of the clinical and laboratory involvements in CTX.

## Competing interests

The authors declare that they have no competing interests.

## Authors' contributions

All authors have read and approved the final manuscript. SFC had substantial contributions to conception and design, data acquisition and analysis, drafting the manuscript and revising the manuscript. NWT, CCC, CHL, CRH and YCC had substantial contributions to conception and design, clinical data analysis. WNC had substantial contributions to conception and design, data analysis, critical revision and final approval of the revision.

## Pre-publication history

The pre-publication history for this paper can be accessed here:

http://www.biomedcentral.com/1471-2377/11/63/prepub
